# An interesting presentation of invasive bladder carcinoma as pseudo renal failure

**DOI:** 10.1590/2175-8239-JBN-2019-0127

**Published:** 2020-12-18

**Authors:** Ashwin Shekar, Anuj Dumra, Dinesh Reddy, Hardik Patel

**Affiliations:** 1 Sri Sathya Sai Institute of Higher Medical Sciences, Department of Urology, Prashantigram, Puttaparthi, Andhra Pradesh 515134, India.

**Keywords:** Renal Insufficiency, Carcinoma, Squamous Cell, Urinary Bladder, Insuficiência Renal, Carcinoma de Células Escamosas, Bexiga Urinária

## Abstract

Ascites and oliguria with an increasing serum creatinine level are often observed in patients with acute renal failure. However, these symptoms are also noted in individuals with intraperitoneal urinary leakage and can be mistaken for acute renal failure. This rise in creatinine in such patients is called pseudo renal failure and it happens by a process of reverse peritoneal dialysis. In literature, the most commonly described condition that leads to this clinical picture is following a spontaneous or missed bladder perforation. We, herein, report a case of carcinoma of the bladder that presented with features resembling acute renal failure, which later turned out to be pseudo renal failure due to intraperitoneal urinary extravasation from a forniceal rupture. The patient was managed with emergency with a percutaneous drain followed by a percutaneous nephrostomy, which led to normalization of creatinine. Cystoscopy revealed the bladder growth in an intact small capacity bladder and biopsy confirmed it as a muscle invasive squamous cell carcinoma. Due to advanced nature of his malignancy, he underwent a palliative ileal conduit diversion but he later developed chest metastasis and ultimately succumbed to the disease. Intraperitoneal urinary leakage due to forniceal rupture presenting as pseudo renal failure is a rare presentation of carcinoma bladder. Sudden onset abdominal discomfort, increasing ascites, hematuria, and oliguria with elevated renal parameters needs consideration and exclusion of this entity. The diagnostic dilemma associated with this rare presentation along with the management and prognosis in such patients of carcinoma bladder are discussed.

## Resumo

Ascite e oligúria com um nível crescente de creatinina sérica são frequentemente observadas em pacientes com insuficiência renal aguda. Entretanto, esses sintomas também são notados em indivíduos com extravasamento urinário intraperitoneal e podem ser diagnosticados como lesão renal aguda erroneamente. Este aumento de creatinina em tais pacientes é chamado de pseudo insuficiência renal e ocorre por um processo de diálise peritoneal reversa. Na literatura, a condição mais comumente descrita que leva a este quadro clínico se dá após uma perfuração vesical espontânea ou perdida. Relatamos aqui um caso de carcinoma de bexiga que apresentou características semelhantes à insuficiência renal aguda, e mais tarde se revelou uma pseudo insuficiência renal devido a extravasamento urinário intraperitoneal de uma ruptura de fórnice renal. O paciente foi tratado com emergência com um dreno percutâneo seguido por uma nefrostomia percutânea, que levou à normalização da creatinina. A cistoscopia revelou o crescimento da bexiga em uma bexiga intacta, de pequena capacidade e que a biópsia confirmou como um carcinoma escamoso invasivo muscular. Devido à natureza avançada de sua malignidade, ele foi submetido a um desvio de conduto ileal paliativo, mas posteriormente desenvolveu metástase torácica e acabou sucumbindo à doença. O vazamento urinário intraperitoneal devido à ruptura do fórnice renal que se apresenta como pseudo insuficiência renal é uma apresentação rara do carcinoma vesical. Desconforto abdominal de início súbito, ascite crescente, hematúria e oligúria com elevados parâmetros renais precisam de consideração e exclusão desta entidade. O dilema diagnóstico associado a esta rara apresentação, juntamente com o controle e prognóstico em tais pacientes de carcinoma vesicular, são discutidos.

## Introduction

Ascites, elevated creatinine, and decreased urine output in a carcinoma bladder patient is usually suggestive of acute renal failure. However, sometimes this exact presentation can be mimicked in patients who have intraperitoneal leakage of urine and undergo absorption of creatine into the blood stream through a process of reveres auto-dialysis, being called pseudo renal failure.^1^^,^ 2 This rare clinical presentation has been reported in cases of bladder carcinoma following intraperitoneal perforation due to extensive malignant permeation of the wall.^3^ Herein, we report the case of a patient with squamous cell carcinoma of the urinary bladder who presented acutely with rapid onset of abdominal distension with markedly elevated, creatinine which was later proven to be pseudo renal failure caused by extravasation from acute unilateral ureteric obstruction. To the best of our knowledge, this is probably the first reported patient with bladder carcinoma who presented with a clinical picture of pseudo renal failure without a bladder perforation.

## Case report

**Figure 1:**
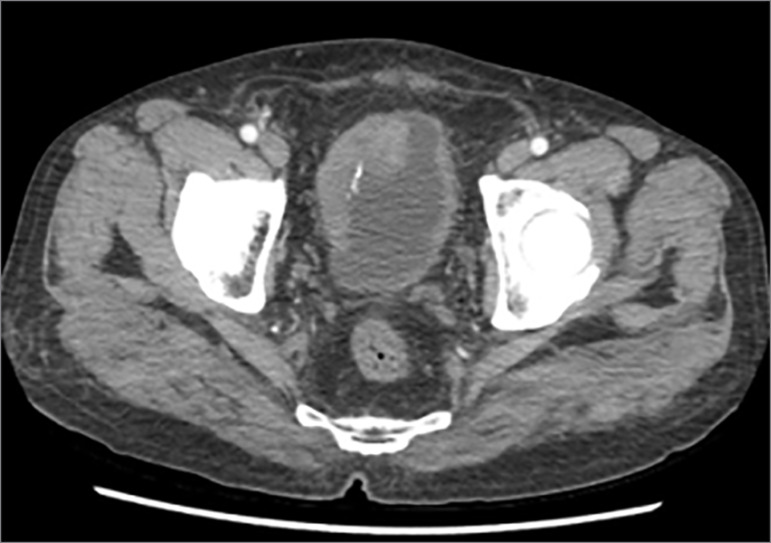
Initial contrast enhanced computed tomography image showing diffuse asymmetric thickening of bladder wall with speck of calcification suggestive of bladder malignancy.

**Figure 2:**
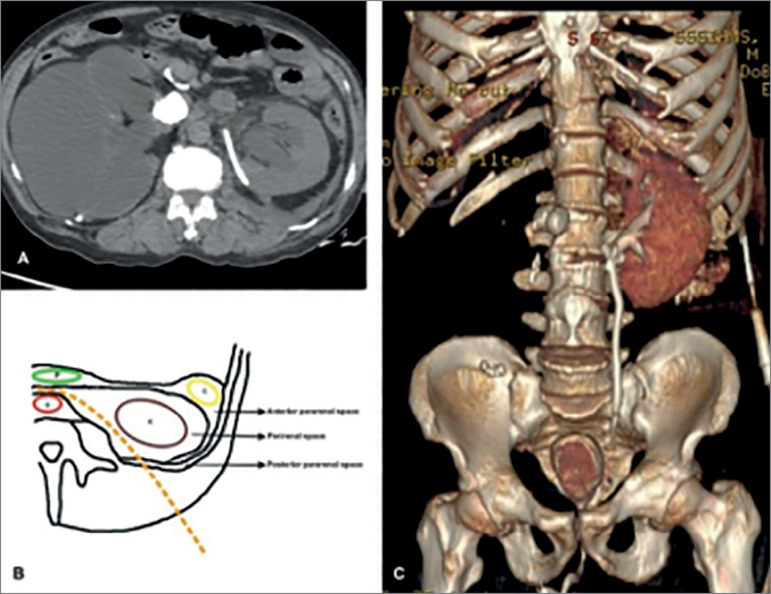
**A.** Axial cut of repeat CT abdomen showing pigtail catheter in left perirenal space. **B.** Diagrammatic representation showing the course of the pigtail catheter (dotted orange line) in the left perinephric space. P-Pancreas; K-Kidney; C-Colon; A-Aorta. **C.** 3D reconstructed image of CT cystogram and nephrostogram showing small intact bladder with no perforation with right distal ureteric obstruction.

A 63-year-old man presented to our outpatient department with history of frequent hematuria. Physical examination was unremarkable and his blood work-up was normal with serum creatinine of 1.2 mg/dL. A contrast-enhanced computed tomography (CT) of the abdomen revealed an extensive infiltrative bladder growth, more on the right lateral wall and dome (Figure 1) with moderate left hydroureteronephrosis and a right poorly functioning kidney due to an obstructing pelviureteric junction stone. He was planned for a diagnostic cystoscopy with a transurethral biopsy. However, he presented to the emergency before his scheduled admission date with hemodynamic instability, abdominal distension, and difficulty in breathing for which he needed ventilatory and inotropic support. His biochemistry panel showed high creatinine (20 mg/dL), hyperkalemia (9 mEq/L), and hyponatremia (128 mEq/L), and his blood gas analysis showed severe metabolic acidosis (pH 7.1). On further questioning of the patient’s caretakers, a history of oliguria with gradually increasing abdominal distension for the past 2 days for which he had undergone tapping of “ascitic” fluid in an outside center was elicited. Anti-hyperkalemic measures were started. A bedside ultrasound showed left hydroureteronephrosis with gross right hydroureteronephrosis and free fluid in abdomen. In view of hemodynamic instability and unavailability of in-house hemodialysis in our setup, decision for an emergency bedside nephrostomy insertion into the solitary functioning left kidney was made. Under ultrasound guidance, bedside percutaneous nephrostomy (PCN) insertion was done and the patient’s general condition improved. There was an initial diuresis with drop in creatinine level to a nadir of 2 mg/dL. Because of persistently elevated creatinine levels, CT imaging was repeated after 4 days of nephrostomy placement and to our surprise, we noticed that the tip of the pigtail catheter was in the left perinephric space (Figure 2A and B) acting as a percutaneous drain with complete resolution of free fluid in the abdomen. The chronology of events of an extremely high initial serum creatinine which had decreased dramatically following drainage of extravasated fluid, fitted into a clinical picture of pseudo renal failure due to absorption of extravasated urine. We hypothesized that the source of the extravasated urine was most probably due to a forniceal rupture due to high pressure obstruction on the left side. In view of the still elevated serum creatinine and the gradually decreasing pigtail catheter output, a PCN was placed within the collecting system under fluoroscopic guidance following which his creatinine normalized. A subsequent CT nephrostogram with cystogram showed a left distal ureteric obstruction and a small, intact bladder, ruling out a bladder perforation (Figure 2C). A cystoscopic biopsy of the bladder growth confirmed it as a muscle invasive squamous cell carcinoma. We explained the diagnosis and prognosis to the patient and proceeded for an explorative laparotomy after 2 weeks of the biopsy. Intraoperatively, it was noted that there was dense desmoplastic reaction around the bladder, which was shrunken and plastered to the pelvic side walls and the pubic bone. Due to the inoperable nature of the disease, plan of radical cystectomy was abandoned and a palliative ileal conduit diversion with right sided nephrectomy was done. The patient did well postoperatively and was discharged after 5 days. However, he developed multiple chest metastasis over the next few months and his general condition deteriorated steadily, ultimately resulting in his death at 3 months following surgery due to cachexia.

## Discussion

Pseudo renal failure is a condition characterized by a status of laboratory abnormalities of acute kidney injury in the setting of previous normal kidney function from urinary ascites.^1^ The new onset of renal failure in such clinical cases is based on the concept of “reverse auto-dialysis” of the peritoneal membrane, a reverse form of continuous peritoneal dialysis that is most apparent when the presentation is delayed.^2^ The expression ‘reverse peritoneal dialysis’ was first used in 1991.^3^ It is characterized by a flux of small molecules such as creatinine and urea from urine collected in the peritoneum along a concentration gradient opposite to conventional peritoneal dialysis where small molecules move from the blood to peritoneal cavity.^4^ As a result, the blood values of creatinine and urea are elevated mimicking renal failure despite normally functioning kidneys. Pseudo renal failure usually appears within 24 hours of urinary extravasation.^5^ In early stages, the serum creatinine levels are usually very high. However, the other renal parameters are surprisingly normal despite a very high creatinine. The rapid early rise in creatinine suggests peritoneal urinary resorption rather than true acute renal failure and some authors believe this to be a diagnostic marker.^5^ The more the delay in presentation and diagnosis, the more severe the biochemical abnormalities. In later stages it manifests as elevated serum urea, creatinine and potassium, and low serum sodium, with development of a metabolic acidosis.

In adulthood, the commonest cause of such a presentation is the delayed diagnosis of a spontaneous or traumatic bladder perforation.^6^^-^ 10 Bladder cancer is one such rare cause of spontaneous bladder perforation that can lead to this clinical picture.^11^^-^ 19 Interestingly, most of the cases of bladder cancer who had spontaneous bladder perforations were in fact, squamous cell carcinomas, like our index case, probably denoting more extensive permeation of wall.^15^^,^ 16 ^,^ 18 ^,^ 19 However, to the best of our knowledge, there are no reports of patients with bladder cancer presenting with a clinical picture of pseudo renal failure, without an obvious bladder perforation. The probable mechanism for this phenomenon in our case may be a peri-renal urine extravasation from a rupture of the calyceal fornix secondary to high intra-pelvic pressures, resulting in urinary ascites followed by subsequent re-absorption by lymphatic or venous channels.^20^ Clinicians should distinguish pseudo renal failure from true renal failure based on laboratory analysis of peritoneal fluid, which has higher levels of urea, creatinine, and potassium than serum.^1^^-^ 10

Emergency management of these patients depend on the clinical scenario. Unfortunately, most of these patients are elderly and are moribund at presentation, thereby precluding any major definitive surgery like a radical cystectomy. If there are signs of peritonitis suggestive of an intraperitoneal rupture, the treatment should be an emergency laparotomy with closure of the rent. 12 ^-^ 19 In patients with extra-peritoneal perforation or a forniceal rupture like our patient, drainage of urinary ascites by paracentesis or pigtail drainage and prompt urinary diversion in the form of a percutaneous nephrostomy would help in normalizing the creatinine and avoiding immediate laparatomy.^11^ Patients with pseudo renal failure rarely require hemodialysis, although it has been reported. Unnecessary renal replacement therapy can be avoided with prompt diagnosis and proper emergency management. However, prognosis in these patients is poor with most of the patients never proceeding to definitive radical cystectomy and succumbing to the disease within a year of presentation.^11^^-^ 19

To conclude, patients with bladder cancer presenting with an acutely rising serum creatinine level, ascites, and oliguria should be assessed for urine leakage by the treating urologist. Early recognition and diversion, as opposed to dialysis therapy, are warranted in such clinical scenario. The key to diagnosis is awareness of the clinical entity and it should be considered in clinically relevant situations, including patients with acute unilateral obstructions. Although diagnosis and early intervention can salvage such patients presenting in emergency setting, long term prognosis of these patients is still poor.
